# Proposed layout of an online store website based on the mental model of Iranian Users

**DOI:** 10.34172/hpp.025.44259

**Published:** 2025-12-30

**Authors:** Ali Akbar Keikha Moghaddam, Iman Dianat, Mohammad Asghari Jafarabadi

**Affiliations:** ^1^Department of Occupational Health and Ergonomics, Faculty of Health, Tabriz University of Medical Sciences, Tabriz, Iran; ^2^Road Traffic Injury Research Center, Tabriz University of Medical Sciences, Tabriz, Iran; ^3^Cabrini Research, Cabrini Health, Malvern, VIC, 3144, Australia; ^4^School of Public Health and Preventative Medicine, Monash University, Melbourne, VIC, 3004, Australia

**Keywords:** Cognitive ergonomics, Mental model, User experience, User performance, Website design

## Abstract

**Background:**

Users’ interaction with the websites shapes their mental model in relation to the website layouts. Consequently, designing websites based on the users’ mental models can lead to better performance and greater user satisfaction. This study aimed to determine the mental model of a sample of Iranian users regarding the object layout of an online store website.

**Methods:**

A total number of 171 participants took part in this study. They were asked to arrange the objects of an online store website including horizontal and vertical menus, search, logo, home, shopping cart, sign-up/login, contact us/about us/help on a Word document page based on their preferences and expectations. Data were analyzed based on the users’ mental models and their demographic details.

**Results:**

Participants located the website objects as follow: simultaneous use of horizontal and right vertical menus (36.3%), search box in the upper-middle area (48%), logo in the upper-middle area (38.6%), home object in the upper-right area (36.3%), shopping cart location in the upper-left area (38%), sign-up/log in in the upper-right area (41.5%), and contact us/about us/help in the upper-right area (28.8%). No significant difference was found between the positions of objects and gender or internet experience, except for the dominant hand with vertical menu and home position.

**Conclusion:**

Difference between the findings of this study and those reported in other countries may suggests that the written language can influence the position of certain website objects. It is, therefore, may not be feasible to utilize the results of studies from other countries in the design of domestic websites.

## Introduction

 Cognitive ergonomics focuses on understanding human cognitive abilities and limitations, to improve the design process and enhance user experiences with technology. Given the large number and diversity of users, website design requires an understanding of users’ cognition, goals and constraints as well as their use of technology.^1^ Mental models are cognitive representations of the real world, first introduced by Kenneth Craik, suggesting that individuals have a mental model of how the world functions on a small scale.^2^ Mental models are mechanisms through which humans can describe the structure and purpose of a system, explain its functioning and states, and predict future system states.^3^ A mental model is knowledge about a system, especially how it works, whether it is a digital system like a website or a physical system. It constructs a model of how a system operates and apply it to new situations that involve a similar system.^4^

 As internet users browse the web, they also construct mental models of website structures and navigation. Consequently, designing a website that aligns with the users’ mental model is likely requires less time for them to become familiar with the website. This allows users to make more informed navigation decisions, leading to a more positive user experience.^5^ Websites that are designed in accordance with the user expectations are considered effective because customers can find the answers they need in the places they expect, which increases the likelihood of users returning to the website.^6^

 Today, the importance of online commerce is undeniable, prompting both large and small companies to continually seek ways to attract new online customers. However, there are still obstacles such as finding a product and confusion between the customer and the online store. Therefore, a key advantage for online stores is placing essential website objects such as shopping cart, menus, help, etc., where most users expect them to be, leading to improved performance.^7^ In fact, Jones and Dumas argue that “knowing what we’re looking for is not enough; we must know where to look for it”.^8^

 On the other hand, the concept of mental models has found its place in several guidelines for designing user interfaces and websites (e.g., Apple Inc. 2007, 2008; IBM and International Standards Organization 1998). These design guidelines emphasize the importance of reflecting users’ mental models, meaning understanding users’ expectations. As a result, by anticipating user habits, it is possible to prevent errors and enhance the efficiency of interactions.^9^

###  Factors influencing mental models

 A number of factors such as culture, language, internet experience, gender, and the type of website may influence the formation of users’ mental models during interactions with websites.^9-14^

 Most web-based applications consider a one-size-fits-all model (North American model), while individuals from different cultures interact and communicate based on their cultural backgrounds, and this model may not necessarily be consistent with the needs of people from other cultures. People from different cultures use different web interface methods and have different mental models for visual representations, navigation, interaction, and layout, with different expectations and communication patterns.^10^ Baharum et al. conducted a study to determine the positioning of website objects according to users’ mental models in 10 Asian countries, and the cross-cultural perspective revealed clear patterns of differences in access to information at any given time and place, emphasizing the importance of website user interface design based on the user’s perspective.^11^ The authors also suggested that in a country with a different culture, websites should be designed not only based on their language but also with consideration of localization in that country.^11^ On the other hand, Bourges-Waldegg and Scrivener stated that existing cultural models are often overly general and clichéd for user interface design, lacking usability tests to validate their claims.^12^

 Different mental models can be explained on the one hand by factors such as the development of the Internet (e.g., expertise), and on the other hand, by demographic factors of users such as age, gender, and level of experience that may affect mental models and expectations.^9^ Experience or even expertise in web page design can change a person’s perspective on the web. Chevalier and Kicka examined the search strategies of novice users, experienced users, and professional web designers and found that website designers were unable to predict and behave like novice users’ strategies effectively.^13^ Roth et al found a significant difference in computer and internet skills and the number of times of internet use between women and men, but the mental model was similar in both genders and between two groups of regular users and website designers. In addition, users generally agreed on the position of most objects of all three types of websites.^9^ Bernard examined the layout of the online store website in experienced and inexperienced users when using the Internet and reported that the layout for website objects was the same in both groups.^14^

 Written language can also be a factor in web design. In mental model studies, where the written language of the participants was from left to right,^9,11,15-19^ users placed the vertical menu on the left side. It seems that in countries with right-to-left writing, such as Persian and Arab countries, there is a tendency to place the menu on the right side. Salmeron et al conducted a study among Arab users with two hypotheses for the position of the website menu; one was based on people’s previous experiences and the other was based on language. They selected two groups of users (native Arabic speakers and those who spoke English) with two types of website presentation (Arabic and English languages) either on the right or left side and found that individuals had a more positive judgment towards Arabic websites with menus on the right side.^20^

 To the authors’ knowledge, there is limited research on website design attributes among Iranian users, who have different reading and writing directions compared to English-speaking users. To date, no study has examined website layout attributes comprehensively to provide a more detailed comparison with English-language studies. As mentioned above, only one study has addressed the position of a vertical menu—specifically whether it is on the right or left side—among a limited number of Arabic-speaking users. This highlights the importance of conducting further research with larger sample sizes among users who read and write from right to left such as Persian language users. Studies to be conducted on this issue would enable a more accurate comparison between their mental models with those of English-speaking users, who have a different sequential flow of the writing system (e.g., reading and writing direction). The findings would also, firstly, serve as a valuable resource for designers of Persian websites. Secondly, comparing these findings with English-language studies will reveal which website elements are most influenced by reading and writing direction. This insight is crucial for web designers and user experience specialists to consider reading and writing direction when creating websites tailored to the culture and language of each country.

 Based on the above mentioned background, the findings of studies in other countries with different culture and language may not be generalizable for the design of websites worldwide. This is an issue that needs further investigation and research in every country, particularly in non-English language countries. Therefore, the present study was conducted to determine the mental model of a sample of Iranian users with regard to the objects layout of an online store website. Findings form research in this area can provide further evidence to develop website design guidelines based on the mental models of users with different languages and cultures.

## Methods

###  Participants 

 A total of 171 participants (65 males and 106 females) volunteered to participate in this study. They were students/staff from the Tabriz University of Medical Sciences (TUMS). Having a minimum of one year of experience of working with the internet was considered as inclusion criteria for the study. The age of participants ranged from 18 to 51 years, with an average ( ± Standard deviation [SD]) of 27.56 ( ± 9.26) years. All participants signed a written consent form before participation and the study protocol was approved by the ethical review committee of the TUMS (code: IR.TBZMED.REC.1398.369).

###  Setting and procedure 

 The study was conducted in a computer room within the TUMS. Prior to the study, all participants received information about the study aims and objectives as well as instructions on how to create their mental model for an online store website. Data were collected in two stages. Demographic information including gender, dominant hand, and experience of using internet (year) were collected in the first stage. In the next stage, the optimal placement of objects of an online store website using the users’ mental models was determined. This was performed during a 10-minute session dedicated to create the preferred layout of the online store by each participant. For this, the objects of an online store website, including the logo, shopping cart, search box, home, help, about us, contact us, sign-up/login, horizontal menu, vertical menu, and website content, were placed as images in a Word document (Figure S1). The Word document was set to A4 size in portrait orientation, and the page was zoomed in to fit the entire monitor screen (e.g., dimensions of the Word page was equal to the monitor screen). Participants were then asked to arrange the website objects on the full-page Word document based on their expectations. They were also informed that they could adjust the size of each object such as the logo, search box, etc., according to their preferences. With regard to the horizontal and vertical menus, participants had the choice to use either both menus or only one of them, and if they did not wish to use either of them, they could remove it. The session output was a Word file which was saved as a PDF file for subsequent analyses.

###  Data analysis

**Figure 1:**
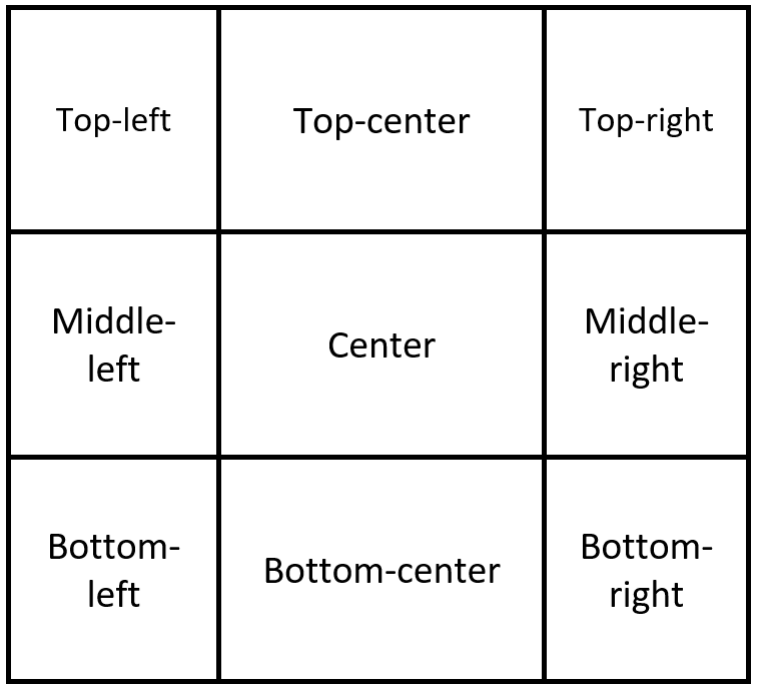
Regions for the website objects

 To determine the location of the website objects, the A4 size pages containing the website layouts created by the participants were divided into 3 × 3 = 9 regions (as shown in Figure 1). Descriptive (frequency and percentage) and analytical statistics were used for the data analysis. Differences in the website object location based on the demographic details of the participants were examined by the one-way ANOVA analysis. *P* ≤ 0.05 was set for the statistical analyses. SPSS v.23 was used for the data analysis.

## Results

###  Menu

Table 1 shows the results of study regarding the position of the horizontal and vertical menus. Sixty-two (36.3%) participants chose to use both horizontal and vertical (right) menus simultaneously, while 39 (22.8%) participants chose only the vertical menu on the right as their priority for the menu position.

**Table 1:** Results of the website objects location

**Website object**	**Frequency (%)**	**Gender**		**Dominant hand**		**Internet experience**		
		**Male**	**Female**	**Right**	**Left**	**1–5**	**6–10**	**>10**
Menu								
Horizontal with right-vertical	62 (36.3)	32	30	60	2	10	27	25
Only right-vertical	39 (22.8)	12	27	38	1	13	13	13
Horizontal with left-vertical	32 (18.7)	8	24	28	4	11	12	9
Only horizontal	26 (15.2)	10	16	25	1	9	8	9
Only left-vertical	9 (5.3)	3	6	9	0	1	5	3
Only middle-vertical	3 (1.8)	0	3	1	2	1	1	1
Total	171 (100.0)	65	106	161	10	45	66	60
Search box								
Middle-up	82 (48)	27	55	77	5	19	31	32
Left-up	59 (34.5)	27	32	56	3	15	25	19
Right-up	23 (13.5)	9	14	22	1	22	1	23
Full page	7 (4.1)	2	5	6	1	6	1	7
Total	171 (100.0)	65	106	161	10	45	66	60
Logo								
Middle-up	66 (38.6)	28	38	65	1	15	28	23
Right-up	59 (34.5)	22	37	53	6	17	21	21
Full page	27 (15.8)	9	18	25	2	7	11	9
Left-up	19 (11.1)	6	13	18	1	6	6	7
Total	171 (100.0)	65	106	161	10	45	66	60
Home								
Text-right-up	62 (36.3)	23	39	61	1	14	23	25
Both-right-up	47 (27.5)	17	30	43	4	12	19	16
Image-right-up	39 (22.8)	20	19	37	2	7	18	14
Text-left-up	6 (3.5)	3	3	4	2	4	0	2
Image-left-up	6 (3.5)	1	5	5	1	2	3	1
Both-middle-up	3 (1.8)	0	3	3	0	2	0	1
Text-middle-up	3 (1.8)	1	2	3	0	1	1	1
Both-left-up	3 (1.8)	0	3	3	0	2	1	0
Image-middle-up	2 (1.2)	6	13	18	1	1	1	0
Total	171	65	106	161	10	45	66	60
Shopping cart								
Left-up	65 (38.0)	27	38	61	4	14	25	26
Right-up	55 (32.2)	25	30	52	3	14	25	16
Middle-up	29 (17)	8	21	27	2	9	9	11
Bottom of page	13 (7.6)	3	10	12	1	3	5	5
Middle-left	6 (3.5)	2	4	6	0	2	2	2
Middle-right	3 (1.8)	0	3	3	0	3	0	0
Total	171	65	106	161	10	45	66	60
Sign-up/Login								
Right-up	71 (41.5)	29	42	65	6	20	23	28
Left-up	63 (36.8)	25	38	61	2	15	25	23
Middle-up	33 (19.3)	9	24	31	2	9	15	9
Bottom of page	2 (1.2)	1	1	2	0	1	1	0
Middle-right	1 (0.6)	1	0	1	0	0	1	0
Middle-left	1 (0.6)	0	1	1	0	0	1	0
Total	171	65	106	161	10	45	66	60
Contact/About us/Help								
Horizontal-right	39 (22.8)	15	24	36	3	4	17	18
Vertical-right	37 (21.6)	14	23	35	2	13	10	14
Horizontal-middle	30 (17.5)	5	25	30	0	7	15	8
Bottom	22 (12.9)	10	12	21	1	9	8	5
Horizontal-left	22 (12.9)	11	11	21	1	5	9	8
Vertical-left	21 (12.3)	10	11	18	3	7	7	7
Total	171	65	106	161	10	45	66	60

###  Search box

 Almost half of the participants (48%) determined the location of the search box in the upper-middle area and 34.5% chose the upper-left area for the search box location (Table 1).

###  Logo

 As shown in Table 1, the upper-middle area (38.6%) and the upper-right area (34.5%) were the most favorable locations expressed by the participants for the logo location.

###  Home

 The home object was examined as both text and graphic icon formats in this study. Sixty-two (36.3%) participants chose only the text format in the upper-right area, and forty-seven (27.5%) chose both the text and graphic icon formats in the upper-right area, and 39 (22.8%) chose only the upper-right location (Table 1). In total, 86.6% of the participants preferred the upper-right area for the home object location (either as text, graphic icon or both).

###  Shopping cart

 The results of study regarding the location of the shopping cart object (shown in Table 1) indicted that sixty-five (38%) participants chose the top-left area for the shopping cart location, followed by the top-right area with 32.2% as the most favorable locations for the shopping cart locations.

###  Sign up/log in

 The upper-right area with 41.5% was determined as the best location for the sign up/log in object, followed by the upper-left area with 36.8% as the second favorable location for this object (Table 1).

###  Contact us/About us/Help

 Although these three objects of the website were presented separately, but all of the participants placed these three objects next to each other and only the form of placing them together was different in terms of horizontal or vertical. According to the results of the contact us/about us/help objects (Table 1), a higher percentage of participants (22.8%) placed these three objects together horizontally on the upper-right area and 21.6% chose these three options vertically on the right side.

###  Relationships between the website object location and demographic details

 The results of univariate ANOVA analysis showed significant difference in the location of menu (F = 6.33; *P* < 0.05) according to the dominant hand of the participants. This finding indicated that the right handed participants generally preferred to use both horizontal and vertical (right) menus simultaneously, while the left- handed participants preferred simultaneous horizontal and vertical (left) menus (Table 1). There was also a significant difference in the home objects between the right- and left-handed participants (F = 5.47; *P* < 0.05), so that the right-handed participants generally preferred only the text format in the upper-right area, whereas the left-handed participants preferred both the text and graphic icon formats in the upper-right area (Table 1). No other significant difference was found for other demographic details such as gender and internet experience (Table 2).

**Table 2:** Differences in the website object location based on the demographic details of participants

	**Menu**	**Search box**	**Logo**	**Home**	**Shopping cart**	**Register/Login**	**Contact us/About us/Help**
Gender	NS	NS	NS	NS	NS	NS	NS
Dominant hand	*P* < 0.05	NS	NS	*P* < 0.05	NS	NS	NS
Internet experience	NS	NS	NS	NS	NS	NS	NS

NS = not significant.

###  Optimal layout of the website objects

**Figure 2:**
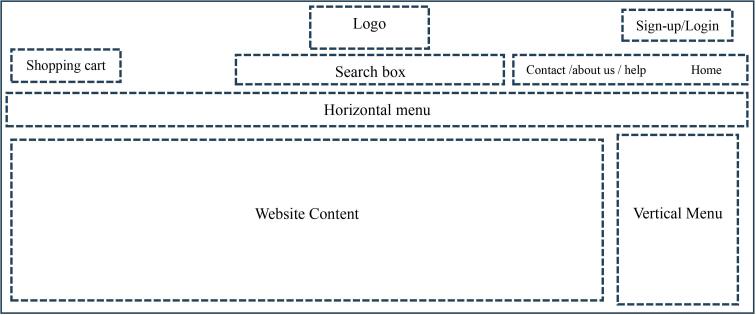
Optimal layout of an online store based on the users’ mental model

 Based on the results of the users’ mental model for the website object location, the best layout for the objects of an online store was can be proposed as follows (Figure 2).

## Discussion

 The aim of the present study was to determine the mental model of a sample of Iranian users in relation to the objects layout of an online store website. The findings show that the position considered by the Iranian users for some website objects is completely different from the findings of the foreign (English language) studies in this field, which can be contributed to the different written language in these studies.

 Compared to the similar studies in this field, our findings demonstrated that individuals’ mental models concerning website layout are notably influenced by the written language. For instance, in countries where the written language reads from left to right, objects like the logo, home, and vertical menu are commonly positioned on the left side.^9,11,17,21^ Considering this issue, it can be stated that the written language is an important factor in determining the position of website objects in any country. This is an aspect that the web and digital product designers should take into account. The results also showed the impact of written language, so that the majority of participants preferred the right side for the vertical menu, which is similar to the findings of Salmeron et al^20^ among the Arabic language users. This finding can be attributed to this fact that although the Arabic language is a foreign language for the Iranians, the sequential flow of the writing system for both languages (Persian and Arabic) is right to left, while this is left to right for English.

 Other differences were also found between our results and those of foreign studies. In similar foreign studies, the website logo and home objects were typically positioned on the left side. In contrast, we found that the logo was centrally located, slightly skewed to the right. Moreover, most participants preferred the home object being placed on the right side. These findings can be, to a large extent, attributable to the impact of written language.

 Shopping cart is one of the most used objects in online store websites. Most participants in this study positioned the shopping cart object on the left-top side, a notable contrast to studies conducted in the left-to-right written language contexts, where it is typically placed on the right-top side. Regarding the website objects analyzed, the vertical menu, logo, home, and shopping cart objects seem to be more influenced by the written language, whereas other objects appear to be less influenced by this factor.

 The investigation of the relationship between the positions of website objects and demographic details of the participants such as gender, dominant hand, and internet experience revealed significant results solely for the dominant hand, with no significant correlation found for gender and internet experience. The lack of relationship of the website object location with gender and internet experience is generally consistent with the findings of previous research.^9^ However, little is known about the relationship between the website object location and the dominant/non-dominant hand of the participants due to limited research in this area. Although there were only 10 left-handed participants in our sample, the results indicated a significant impact of this factor on the placement of the vertical menu, as 40% of the left-handed participants preferred the left side for this object. However, this possibility should be investigated with a larger sample size in future to determine the simultaneous effect of the two factors of written language and dominant hand on users’ preferences in website layout.

## Limitations

 The findings of this study should be interpreted in light of some limitations. One limitation may be a relatively small sample size and testing participants from one of the cities in Iran. Also participants were generally students and employees from the TUMS. Therefore, further studies using a larger sample size and more diverse residents and settings are recommended.

## Conclusion

 This study was conducted on a sample of Iranian users to propose an improved layout for the online store website objects based on their mental models. The results showed differences in the positioning of some website objects such as vertical menu, logo, home, and shopping cart compared to the studies conducted in other countries. This can be attributed to the right-to-left written Persian (Iranian) language compared to the left-to-right written English language. Nevertheless, the lack of relationship between gender and internet experience with the website object layout is generally in agreement with the findings of previous research. The findings suggest that the design of native websites should be based on the expectations and preferences of the targeted community.

## Competing Interests

 There was not conflict of interest.

## Ethical Approval

 This study was approved by the Ethics Committee of Tabriz University of Medical Sciences (Approval ID: IR.TBZMED.REC.1398.369).

## Supplementary Files

Supplementary file contains Figure S1.
