# Supplementary figures and images for: Proposed layout of an online store website based on the mental model of Iranian Users
Source: Health Promot Perspect. 2025 Dec 30;15(4):343–9. doi: 10.34172/hpp.025.44259 (PMC13156283; doi:10.34172/hpp.025.44259)

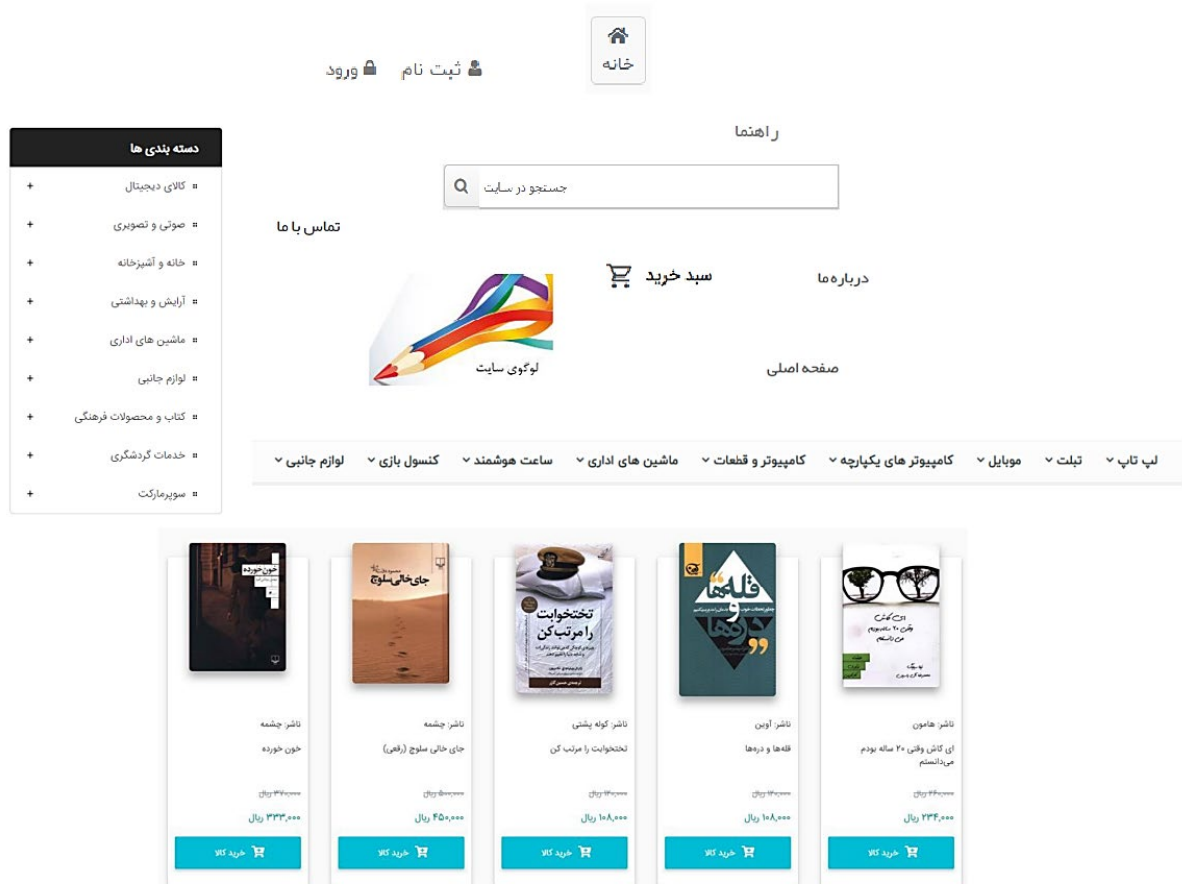

Figure S1. Prototype of the website objects

Supplement: Supplementary file 1 — Supplementary file contains Figure S1. [file hpp-15-343-s001.pdf]
